# Evaluation of fluralaner as an oral acaricide to reduce tick infestation in a wild rodent reservoir of Lyme disease

**DOI:** 10.1186/s13071-020-3932-7

**Published:** 2020-02-13

**Authors:** Jérôme Pelletier, Jean-Philippe Rocheleau, Cécile Aenishaenslin, Francis Beaudry, Gabrielle Dimitri Masson, L. Robbin Lindsay, Nicholas H. Ogden, Catherine Bouchard, Patrick A. Leighton

**Affiliations:** 1 Département de pathologie et microbiologie, Faculté de médecine vétérinaire, Université de Montréal, Saint-Hyacinthe, QC Canada; 2 Groupe de recherche en épidémiologie des zoonoses et santé publique, Faculté de médecine vétérinaire, Université de Montréal, Saint-Hyacinthe, QC Canada; 3 Centre de recherche en Santé Publique, Université de Montréal, Montréal, QC Canada; 4 Département de santé animale, CÉGEP de Saint-Hyacinthe, Saint-Hyacinthe, QC Canada; 5 Groupe de recherche en pharmacologie animale, Département de biomédecine vétérinaire, Faculté de médecine vétérinaire, Université de Montréal, Saint-Hyacinthe, QC Canada; 6 Zoonotic Diseases and Special Pathogens Division, National Microbiology Laboratory, Public Health Agency of Canada, Winnipeg, MB Canada; 7 Public Health Risk Sciences Division, National Microbiology Laboratory, Public Health Agency of Canada, Saint-Hyacinthe, QC Canada

**Keywords:** Lyme disease, Mice, Isoxazolines, Fluralaner, Ticks, *Ixodes scapularis*, *Peromyscus* spp.

## Abstract

**Background:**

Lyme disease (LD) is an increasing public health threat in temperate zones of the northern hemisphere, yet relatively few methods exist for reducing LD risk in endemic areas. Disrupting the LD transmission cycle in nature is a promising avenue for risk reduction. This experimental study evaluated the efficacy of fluralaner, a recent oral acaricide with a long duration of effect in dogs, for killing *Ixodes scapularis* ticks in *Peromyscus maniculatus* mice, a known wildlife reservoir for *Borrelia burgdorferi* in nature.

**Methods:**

We assigned 87 mice to 3 fluralaner treatment groups (50 mg/kg, 12.5 mg/kg and untreated control) administered as a single oral treatment. Mice were then infested with 20 *Ixodes scapularis* larvae at 2, 28 and 45 days post-treatment and we measured efficacy as the proportion of infesting larvae that died within 48 h. At each infestation, blood from 3 mice in each treatment group was tested to obtain fluralaner plasma concentrations (C_p_).

**Results:**

Treatment with 50 mg/kg and 12.5 mg/kg fluralaner killed 97% and 94% of infesting larvae 2 days post-treatment, but no significant effect of treatment on feeding larvae was observed 28 and 45 days post-treatment. Mouse C_p_ did not differ significantly between the two tested doses. Mean C_p_ decreased from 13,000 ng/ml in the 50 mg/kg group and 4000 ng/ml in the 12.5 mg/kg group at Day 2 to < 100 ng/ml in both groups at Day 45.

**Conclusions:**

We provide the first evidence that fluralaner is effective for killing immature ticks in *Peromyscus* mice, a first step in evaluating its potential for treating wild rodents as a public health intervention to reduce LD risk in endemic areas.
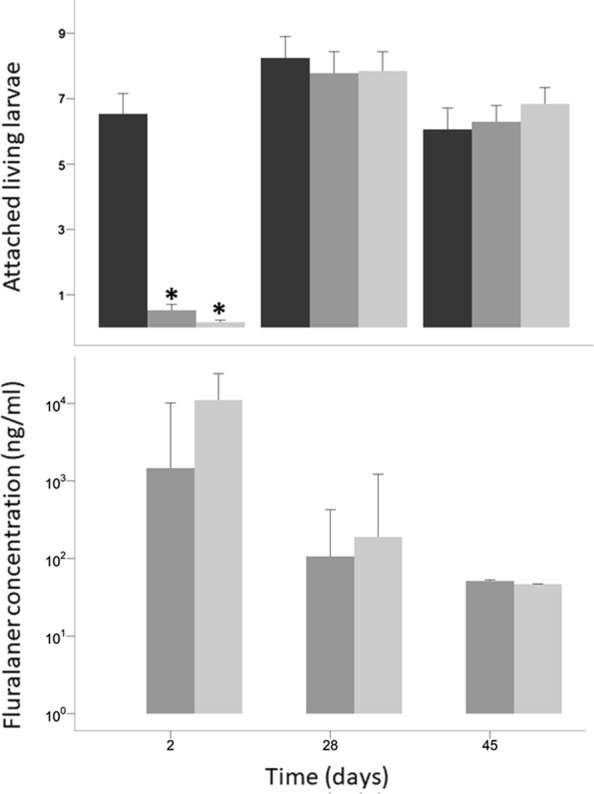

## Background

Lyme disease (LD), caused by the spirochete *Borrelia burgdorferi* [1], is the most important tick-borne disease in Europe and North America [2]. In the USA, the annual incidence rate was 7.2 reported cases per 100,000 people with 33,000 reported cases in 2018 alone [3]. In southern Canada, Lyme borreliosis is currently emerging, associated with the northward spread of the tick *Ixodes scapularis*, with the number of annual reported cases increasing from 144 in 2010 to 2025 in 2017 [4–6]. Because LD is a significant burden for public health, different strategies have been developed to prevent disease transmission to humans, including promoting the adoption of personal preventive measures and reducing tick density in the environment. Tick control measures include the direct application of acaricides in the environment or the treatment of the main tick hosts, such as the white-tailed deer, with oral or topical acaricides [7]. Another potential intervention approach is to treat key reservoirs of *B. burgdorferi*, such as *Peromyscus* spp. mice, to decrease the density of ticks in the environment and/or the prevalence of infection in questing ticks, both of which contribute to the density of infected ticks in the environment which is the main measure of the acarological risk of LD [7–9]. Oral vaccination of mice against *B*. *burgdorferi*’s outer surface protein A (OspA) is reported in the literature as an effective way to reduce the prevalence of the spirochete among host seeking ticks [10, 11]. The application of topical acaricides to wild rodents using treatment stations has also been used to effectively reduce tick density in the environment [12–16].

In 2014, a novel ectoparasiticide family called isoxazolines reached the veterinary drug market. Isoxazolines are non-competitive inhibitors of y-aminobutyric acid (GABA)- and l-glutamate-gated chloride channels (GABACl and GluCl), a target that they share with other ectoparasiticides like fipronil, dieldrin and avermectins [17, 18]. More specifically, isoxazolines mostly act on the GABACl channel by blocking ion channel opening [17–20]. Isoxazolines, like sarolaner and afoxolaner, have been shown to kill adult ticks and prevent *B*. *burgdorferi* transmission in dogs [21, 22]. Fluralaner, another member of this new family, is noted for its ability to kill ticks rapidly and for its long efficacy period following a single oral administration, when used in dogs [23, 24]. Wengenmayer et al. [24] showed that, in dogs, fluralaner (Bravecto^TM^ chewable formulation) killed 98% of infesting adult *Ixodes ricinus* ticks within 24 hours following a single oral administration up to 12 weeks post-treatment. A pharmacology study in dogs supported the clinical observations of a long duration effect by measuring a fluralaner half-life of 12–15 days and a quantifiable plasmatic concentration for up to 112 days [25]. These two characteristics, high efficacy and long duration of effect, are attractive features for treatment of wildlife where providing an effective dose to a significant proportion of the host population can be both difficult and costly. In addition, isoxazolines have been shown to be safe when applied at many times the recommended dose in both mammals (dogs and rats [26–28]) and birds (chickens [29, 30]). Some toxicological data about fluralaner and related compounds like afoxolaner and sarolaner exist for laboratory mice (*Mus musculus*) but they are limited to genotoxicity and mutagenicity [20, 29, 31].

Despite the potential of rodent-targeted interventions for reducing LD risk in the environment and the unique pharmacological properties of fluralaner and other isoxazolines, there are currently no data on the efficacy of this product in mice, and specifically in wild mice of the genus *Peromyscus. Peromyscus* mice are considered to be the primary wildlife reservoirs for *Borrelia burgdorferi* in much of North America [32, 33].

In the present study, we administered fluralaner to *Peromyscus* mice and then infested mice with larval *Ixodes scapularis* ticks in a controlled trial in a laboratory environment as a first step to evaluate the potential of fluralaner, and more broadly the new isoxazoline family of ectoparasiticide drugs, to kill ticks on wild rodents as a public health intervention.

## Methods

### Animals

Eighty-seven healthy *Peromyscus maniculatus* mice from Rocky Mountain Laboratory (Hamilton, MT, USA) were used in this experiment. *Peromyscus maniculatus* is a competent reservoir for *B*. *burgdorferi* and permissive host for *I*. *scapularis*, and closely phylogenetically related to *P*. *leucopus* the primary reservoir for LD in many parts of North America [32]. The group was composed of 40 male and 47 female adult mice (> 1 year-old) with an average weight (± standard deviation, SD) of 20.1 ± 2.7 g. Mice were individually housed in cages with 580 cm^2^ floors, environmental enrichment, commercial food (Charles River rodent diet, Charles River Laboratory, Wilmington, MA, USA) and tap water during the entire experimentation period. All animals were housed in the same room at a temperature between 22–25 °C, a relative humidity between 50–70%, and a 12:12 h light/dark photocycle. Behaviour was visually assessed daily, and mouse weight was assessed during each manipulation. Mice were euthanized at the end of the experiment or when limit points were reached.

### Experimental design

Mice were randomly allocated to three equal groups of 29 animals: one control group and two treatment groups. Each mouse received a 250 mg peanut butter bait: fluralaner (Bravecto^TM^ chewable formulation, Merck Animal Health, Madison, NJ, USA) was mixed with peanut butter baits in the two treatment groups, while pure peanut butter was given to the control group. The first treatment group received a dose of 50 mg/kg, which is 2 times the minimal targeted treatment dose used for dogs, and the second treatment group received a dose of 12.5 mg/kg, which is half the minimal targeted treatment dose for dogs [23, 24]. The 50 mg/kg dose was chosen since we anticipated more rapid clearance of the molecule by *Peromyscus* mice compared to dogs. The 12.5 mg/kg dose was included to evaluate the potential clinical effect of a dose below the targeted range, which is likely to occur under field conditions. Each mouse received their treatment and access to regular food was maintained during the period when baits were deposited in the cages to mimic the context of a natural environmental intervention with food competition. Bait consumption was verified after 24 h to ensure the entire bait had been consumed.

### Infestations

To evaluate treatment efficacy, each mouse was infested with 20 unfed *I. scapularis* larvae at three time points: 2, 28 and 45 days post-treatment. The larvae were hatched from eggs 2 to 3 months before the start of the study and displayed typical host-seeking behaviours at the time of experimental infestations. Groups of mice were infested with larvae of the same age. Infestation was performed by placing larvae on the ears and fur using fine-tipped forceps. To maximise larval attachment, mice were anesthetized (isoflurane 2%) for 1 h during infestation with heater carpets as thermal support and with an injection of subcutaneous fluid (0.5 ml of NaCl 0.9%). At 12, 24 and 48 h post-infestation, mice were visually inspected under anesthesia for a duration of 5 min to count attached larvae. To visually inspect mice, observers followed a systematic inspection procedure: (i) inspection of the ears, head and face; (ii) inspection of the back; and (iii) inspection of the stomach, legs and tail. Observers were blinded to the treatment in order to prevent bias. At 48 h, a sample of remaining attached larvae was removed from each mouse and observed under a binocular microscope to classify them as dead or alive. Larvae showing movement of the legs, movement of the palps and mouthparts, or midgut pulsation were considered alive and larvae expressing none of these behaviours were considered dead. Larvae with no mouthpart during the observation were excluded because the sampling technique was assumed to be the cause of death. The proportion of attached larvae that died was obtained from the observations of larvae and was used to calculate the number of attached living larvae.

### Statistical models

Three generalized linear models (GLMs) were used to analyze the data. The dependent variable for Model 1 was the number of attached larvae. The dependent variable for Model 2 was the number of attached living larvae. Both models 1 and 2 used a negative binomial distribution to account for overdispersion. Independent variables for Models 1 and 2 were the treatment dose, the time elapsed (h) between infestation and larva count, the time elapsed (days) between treatment administration and larva count and mouse sex. Mouse ID was included in both models as a random factor to account for repeated measures. For Model 3, the dependent variable was the proportion of attached larvae on each mouse that were dead at 48 h for each infestation, hereafter termed “mortality proportion”, modelled using a binomial distribution. The independent variables were the treatment dose, the time elapsed (days) between treatment administration and larvae count, mouse sex and mouse ID as random factor. Sex was added as a covariate in all models because a link exists between this factor and the number of ticks infesting small mammals [33]. Model fit was evaluated using Pearson residual plots. Statistical analyses were performed using R version 3.5.1 with *glmmADMB*, *lme4* and *ggplot2* packages [34–38].

### Efficacy assessment

Efficacy was defined as the proportion of larvae killed due to the treatment and was calculated based on the number of attached living larvae according to Abbott’s formula [39]

$${\text{Efficacy }}\left( \% \right) = \frac{{{\text{Mc}} - {\text{Mt}}}}{\text{Mc}} \times 100$$

where Mc is the arithmetic mean of the number of attached living larvae in the control group and Mt is the arithmetic mean of the number of attached living larvae in treatment groups. For all experimental groups, detached larvae were assumed to be dead.

### Concentration of fluralaner in blood

Mouse blood was sampled under anesthesia from the lateral femoral vein on 3 mice in each treatment group on each infestation day, i.e. at Day 2, 28 and 45 post-treatment. Following sampling, the blood was centrifuged at 3000×*g* for 15 min to extract the plasma. Two hundred µl of internal standard solution (100 ng/ml of reserpine in methanol) was added to 50 µl of plasma samples. The sample was quickly vortexed, left to stand for a period of 10 min and then centrifuged at 12,000×*g* for 10 min. The supernatant was transferred into an injection vial for HPLC-MS analysis. The HPLC system was a Vanquish Flex UHPLC system (Thermo Fisher Scientific, San Jose, CA, USA). The chromatography was achieved using a gradient mobile phase along with a microbore column Thermo BioBasic Phenyl (Thermo Fisher Scientific) 50 × 1 mm with a particle size of 5 μm. The initial mobile phase condition consisted of acetonitrile and water (both fortified with 0.1% formic acid) at a ratio of 5:95. From 0 to 1 minute, the ratio was maintained at 5:95. From 1 to 5 min, a linear gradient was applied up to a ratio of 20:80 and maintained for 3 min. The mobile phase composition ratio was reverted at the initial conditions and the column was allowed to re-equilibrate for 7 min for a total run time of 15 min. The flow rate was fixed at 75 µl/min and 2 µl of samples were injected. A Q Exactive Orbitrap Mass Spectrometer (Thermo Fisher Scientific) was interfaced with a UltiMate 3000 Rapid Separation UHPLC system (Thermo Fisher Scientific), using a pneumatic assisted heated electrospray ion source. MS detection was performed in positive ion mode, operating in high-resolution accurate-mass (HRAM) scan mode. Nitrogen was used for sheath and auxiliary gases and were set at 10 and 5 arbitrary units. The heated ESI probe was set to 4000 V and the ion transfer tube temperature was set to 300 °C. The scan range was set to *m/z* 500–700. Data were acquired at a resolving power of 140,000 (FWHM) using an automatic gain control target of 3.0 × 10^6^ and maximum ion injection time of 200 msec. Targeted drug quantification was performed by MS detection using specific precursor masses based on monoisotopic masses (i.e. [M+H]^+^ ions). Quantification was performed by extracting specific precursor ions using a 5 ppm mass window. Instrument calibration was performed prior to all analysis and mass accuracy was notably below 1 ppm using Pierce^TM^ LTQ Velos ESI positive ion calibration solution (Thermo Fisher Scientific) and automated instrument protocol. Fluralaner quantification was performed using peak-area ratio of fluralaner, and the internal standard reserpine and concentrations were determined by interpolating unknowns from the calibration curve constructed with a standard prepared in mouse plasma. The observed precision and accuracy were < 15%. Plasmatic concentrations were statistically analyzed for each time point with the non-parametric Mann-Whitney-Wilcoxon test.

## Results

### Animals and bait consumption

Eight mice died or were euthanized according to the protocol limit points before the completion of the experiment (Table 1). All mice completely consumed the 250 mg bait within the first 24 h after administration.

**Table 1:** Number of attached larvae on mice 48 hours post-infestation for each experimental group at Day 2, Day 28 and Day 45 after treatment administration

Time since treatment (days)	Count of attached larvae						
	Descriptive statistics	0 mg/kg		50 mg/kg		12.5 mg/kg	
		M	F	M	F	M	F
2	Mean^a^	7.0	8.1	3.7	4.6	3.5	3.9
	Range (min–max)	3–13	2–14	1–7	3–11	1–7	2–8
	Total (*n*)^b^	213 (26)		117 (26)		110 (27)	
	Sample^c^	151		92		70	
	Death	19		86		61	
28	Mean^a^	7.6	9.2	10.3	8.9	10.5	8.5
	Range (min–max)	3–15	5–15	6–15	5–13	6–18	4–12
	Total (*n*)^b^	238 (26)		246 (24)		254 (26)	
	Sample^c^	155		138		145	
	Death	4		21		18	
45	Mean^a^	6.3	6.7	7.3	8.1	5.6	7.9
	Range (min–max)	3–9	3–15	5–9	4–15	3–9	1–14
	Total (*n*)^b^	110 (15)^d^		195 (23)		196 (25)	
	Sample^c^	86		175		165	
	Death	4		17		18	

^a^Arithmetic mean

^b^Number of mice contributing to the total tick count

^c^Number of larvae sampled at 48 h post-infestation

^d^11 mice were not tested because of lack of resources

*Abbreviations*: M, male mice; F, female mice

### Attached larvae (Model 1)

**Fig. 1:**
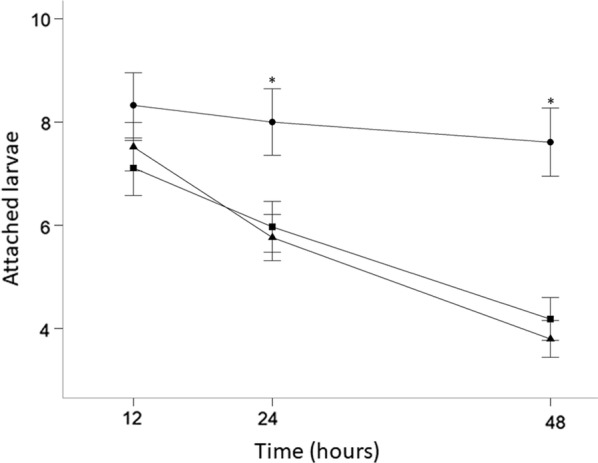
Mean number of attached larvae 12, 24 and 48 h following infestation carried out 2 days after administration of fluralaner treatment. Mice were infested with 20 larvae at time 0 and ticks were counted at 12, 24 and 48 h post-infestation. Error bars are ± 1 SE. *Key*: Circle, 0 mg/kg; square, 50 mg/kg; triangle, 12.5 mg/kg; *, a statistically significant difference compared with the 0 mg/kg group (GLM, Wald-test, *P* < 0.01)

**Fig. 2:**
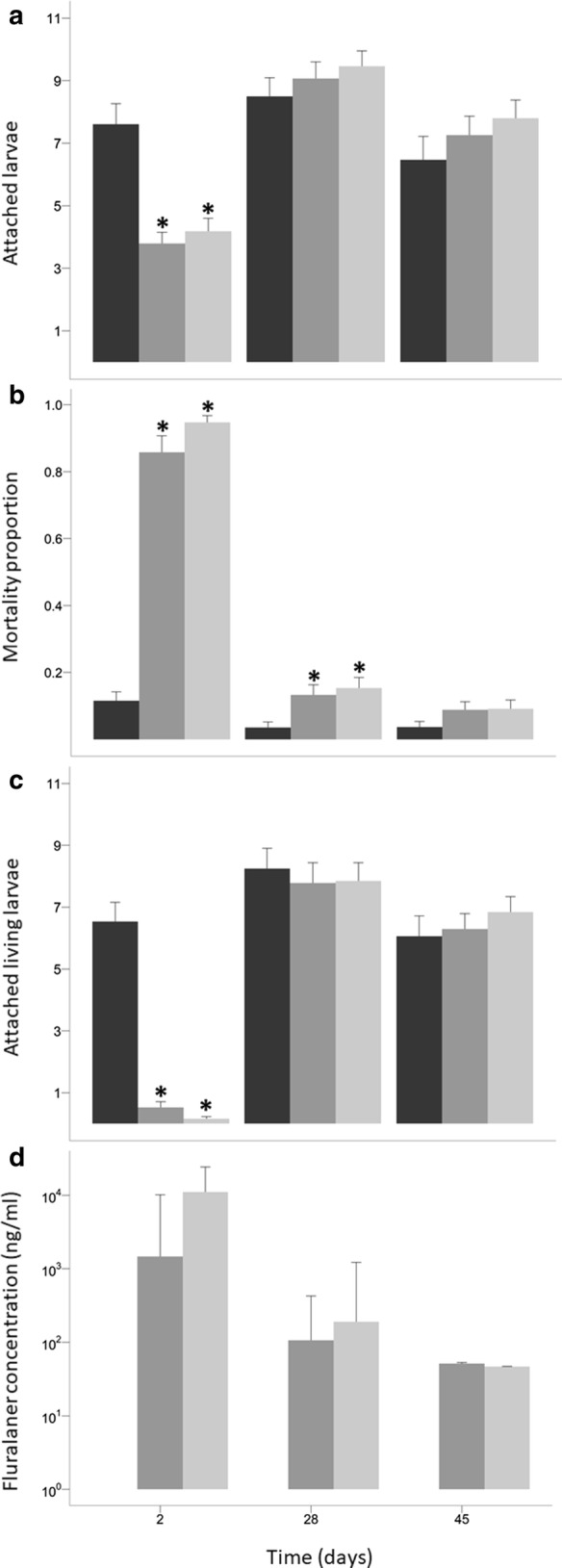
Effect of fluralaner treatment 48 h following infestations carried out at Day 2, 28 and 45 post-treatment. **a** Mean number of attached larvae at 48 h obtained by mouse visual inspections. **b** Mortality proportion of larvae at 48 h post-infestation. A sample of the remaining attached larvae was collected at 48 h and observed under microscope to evaluate if they were dead or alive. **c** Mean number of attached living larvae at 48 h calculated from the number of attached larvae and the larvae mortality proportion. **d** Fluralaner C_p_ arithmetic mean obtained from blood samples of three mice from each treatment group. Error bars represent ± 1 SE in **a**, **b** and **c**, and ± 1 SD in **d**. *Key*: black, 0 mg/kg; dark grey, 12.5 mg/kg; light grey, 50 mg/kg; *, a statistically significant difference compared with the control group (0 mg/kg) (GLM, Wald-test, *P* < 0.01)

The number of attached larvae decreased over the course of the 48 h post-infestation observation period in both treatment and control groups. During the first infestation (Day 2 post-treatment), the number of attached larvae in the two treatment groups significantly decreased from a mean (± standard error, SE) of 7.3 ± 0.4 to a mean of 4.0 ± 0.3 attached larvae between 12 and 48 h post-infestation (GLM, Wald-test, *P* < 0.001). In the control group, the mean number of attached larvae slightly decreased from 8.3 ± 0.5 (SE) to 7.6 ± 0.6 (SE) (GLM, Wald-test, *P* = 0.58). The reduction in the number of attached larvae was significantly higher in the two treatment groups than in the control group (GLM, Wald-test, *P* = 0.001) (Fig. 1). Both treatment groups showed similar reductions of the number of attached larvae (GLM, Wald-test, *P* = 0.92). The treatment effect on the mean number of attached larvae was no longer significant for the Day 28 (GLM, Wald-test, *P* = 0.57) and Day 45 (GLM, Wald-test, *P* = 0.33) infestations (Fig. 2a).

### Mortality proportion (Model 3)

A larger number of attached ticks, dead and alive, was collected in the control group (*n* = 151) in comparison to both treatment groups at Day 2: 92 in the 50 mg/kg group and 70 in the 12.5 mg/kg group. This difference was less pronounced at Day 28: 155 in the control group; 138 in the 50 mg/kg group; and 145 in the 12.5 mg/kg group (Table 1). In total the proportion of attached larvae that died was 93%, 87% and 8% for the 50 mg/kg, the 12.5 mg/kg and the control groups, respectively, at Day 2 after treatment administration. Treatment administration was significantly associated with a high larval mortality proportion (GLM, Wald-test, *P* < 0.001). Mortality proportion decreased significantly over time (GLM, Wald-test, *P* < 0.001) and became statistically non-significant at Day 45 post-treatment (Fig. 2b). The most substantial reduction in mortality proportion occurred between Day 2 and Day 28 (GLM, Wald-test, *P* < 0.001) with no statistical difference between Day 28 and Day 45 (GLM, Wald-test, *P* = 0.2).

### Attached living larvae (Model 2) and efficacy

On Day 2, both treatment groups showed a significantly greater reduction in the number of attached living larvae compared to the control group (GLM, Wald-test, *P* = 0.001). In the 50 mg/kg and the 12.5 mg/kg group the mean number of attached living larvae increased with time since treatment: respectively 0.2 ± 0.1 (SE) and 0.4 ± 0.2 (SE) when ticks attached on Day 2, but 8.0 ± 0.6 (SE) and 7.9 ± 0.6 (SE) when ticks attached on Day 28 (Fig. 2c). On Day 2, fluralaner treatment efficacy was 97% and 94% for the 50 mg/kg and the 12.5 mg/kg groups, respectively. Efficacy decreased at Day 28 to 3% for the 50 mg/kg dose and 4% for the 12.5 mg/kg dose (Table 2).

**Table 2:** Fluralaner dose efficacy at Day 2, Day 28 and Day 45 after treatment administration

Infestation	Dose	Attached living larvae^a^	Efficacy (%)
Day 2	0 mg/kg	6.65	
	50 mg/kg	0.19	97.1
	12.5 mg/kg	0.43	93.5
Day 28	0 mg/kg	8.28	
	50 mg/kg	8.02	3.1
	12.5 mg/kg	7.94	4.1
Day 45	0 mg/kg	6.06	
	50 mg/kg	6.84	0
	12.5 mg/kg	6.30	0

^a^Arithmetic mean

*Note*: Efficacy represents the proportion of ticks killed by the treatment

### Concentration of fluralaner in blood

At Day 2, the plasmatic concentration (C_p_) arithmetic mean (± standard deviation, SD) was 13,815 ± 11,585 ng/ml in the 50 mg/kg group and 4594 ± 6995 ng/ml in the 12.5 mg/kg. Nevertheless, given the great variability in the C_p_ of tested individuals, C_p_ were not statistically different between the two groups (Mann-Whitney U-test, *U*_(6)_ = 7, *P *= 0.4). At Day 28, the differences in C_p_ between treatments decreased with 579 ± 885 (SD) ng/ml in the 50 mg/kg group and 208 ± 277 (SD) ng/ml in the 12.5 mg/kg group (Mann-Whitney U-test, *U*_(6)_ = 7, *P* = 0.4). Plasmatic concentration became roughly the same at Day 45 (Mann-Whitney U-test, *U*_(6)_ = 0, *P* = 0.1) with 46.7 ± 0.5 (SD) ng/ml and 52 ± 1 (SD) ng/ml, respectively (Fig. 2d).

## Discussion

To the best of our knowledge, this study provides the first evidence that fluralaner is effective at killing larval *I. scapularis* ticks feeding upon *Peromyscus* mice. Efficacy two days post-treatment was greater than 90% for both tested doses, suggesting that fluralaner delivered orally using voluntarily-consumed baits has the potential to kill a significant proportion of immature ticks infesting small mammals, thus disrupting the *B. burgdorferi* transmission cycle in nature. While fluralaner did not provide the same duration of high efficacy as seen in dogs, the achieved efficacy of 94% at Day 2 with a 12.5 mg/kg treatment dose indicates that fluralaner provides effective short-term protection in *Peromyscus* mice at a dose 4 times lower than 50 mg/kg and 2 times than 25 mg/kg. Overall, our results suggest that regular administration of fluralaner baits to small mammals during the peak season for immature ticks has the potential to provide a promising new approach for localized reduction of LD risk in North America.

We found that, 2 days post-treatment, fluralaner reduced the mean number of attached larvae on *Peromyscus* mice (Figs. 1, 2a). This suggests that fluralaner treatment at the two doses tested affected larval viability enough to cause them to fall off. This may be associated with an increased susceptibility to host grooming behavior, which is a major factor in explaining mouse ectoparasite infestation rates [40–43], although hard ticks are somewhat resistant to grooming due to their tough cuticles and feeding behaviour which causes them to be anchored to the skin [44]. However, treatment did not bring the number of attached larvae to zero (Fig. 1), even though many attached larvae were in fact dead. The fact that treatment may be effective without causing ticks to detach is an important consideration for the evaluation of treatment efficacy in the absence of a direct evaluation of the viability of larvae. A similar observation was made by Fisara and Webster [45] in their clinical controlled trial of Bravecto^TM^ efficacy in dogs against *Ixodes holocyclus* ticks, in which the authors noted that the presence of attached ticks on dogs could be perceived as a treatment failure but they observed that the remaining ticks were killed by the treatment.

We were able to confirm treatment efficacy by documenting significant tick mortality in attached larvae, which brought the infestation rate based on attached living larvae close to zero in both treatment groups. The significant difference in the proportion of dead larvae was the main observation supporting treatment efficacy at Day 2 post-treatment and was the only significant difference between treatment and control groups at Day 28 (Fig. 2b, c). Unlike a study of fluralaner efficacy against adult *I. ricinus* ticks on dogs, the treatment did not result in an efficacy of 100% within 2 days of treatment administration [24]. This difference could be explained by variability in attachment and the feeding speed of the larvae depending on their ability to bite at the time of infestation, resulting in a delay in the treatment effect [46]. Previously published studies used adult ticks, and the difference in the volume of the blood meals of larvae and adults, could also explain the different results observed in this study [47].

We found that clinical effect of fluralaner bait in mice declined rapidly over time, showing only a marginally greater tick mortality proportion compared to controls, with a limited impact on the attached living larva infestation rate 28 days post-treatment (Fig. 2). This differs from previous findings reported in dogs where fluralaner efficacy against adult ticks remained high for more than 2 months post-treatment [23, 24, 45, 48]. Pharmacokinetics in dogs showed that fluralaner clearance is mainly *via* the hepatobiliary pathway [25, 27]. Systemic clearance of the molecule should be related to hepatic clearance, which is linked to hepatic blood flow [49]. Hepatic blood flow in mice is three times higher (129.6 l/kg/day) than in dogs (44.5 l/kg/day). So this difference, along with other physiological and metabolic differences between dogs and mice may explain the more rapid decline of treatment efficacy observed in the present study [25, 50]. At Day 2 after treatment, C_p_ values in mice for the dose of 50 mg/kg and the dose of 12.5 mg/kg were higher than those seen in dogs at the same doses and the same time point. In contrast, at day 28, mice had a mean C_p_ lower than what Kilp et al. [25] observed in dogs. While faster drug clearance appears to reduce the duration of effect in mice, it may also reduce fluralaner toxicity in mice and increase its therapeutic index in this species.

The C_p_ concentration was highly variable in both treatment groups, particularly shortly following treatment, likely due in part to the oral self-administration of the treatment bait. By 45 days post-treatment, fluralaner concentration decreased below 100 ng/ml (Fig. 2d) also supporting the hypothesis of faster drug clearance in mice than in dogs. In dogs, Kilp et al. [25] measured C_p_ values below the 100 ng/ml threshold just before 60 days or 2 months post-treatment. Similarly, Becskei et al. [48] observed a reduction of the Bravecto^TM^ formulation efficacy in dogs after 60 days. In contrast, we observed the greatest efficacy reduction between day 2 and day 28 post-treatment, with only a marginal effect at 28 days when mean C_p_ values were 578 ng/ml for the 50 mg/kg group and 207 ng/ml for the 12.5 mg/kg group. The absence of difference in clinical effect between 12.5 mg/kg and 50 mg/kg treatment doses is similar to the study of Kilp et al. [25] who found no significant difference in C_p_ area under the curve (AUC) between 12.5 mg/kg and 50 mg/kg doses in dogs. The present study shows no statistical difference in C_p_ for the same dose range at Day 2, 28 and 45 after a single oral administration even with large C_p_ differences between the two groups at Day 2 (Fig. 2d). While this observation correlates with clinical effect, it remains preliminary given the high variability in the C_p_ data and limited statistical power. It is also possible that an efficacy differential between the two doses develops in the shorter term, i.e. somewhere between Day 2 and Day 28 post-treatment, but a greater observation frequency would be required to evaluate this.

The infestation method used in this study resulted in significant loss of larvae between infestation and the observation time points in both treated and control group (Fig. 1). This phenomenon occurred at all infestations and resulted in a low infestation rate at 48 hours for all groups even in the absence of a significant treatment effect (Table 1, Fig. 2a). Grooming behaviour could partly explain this observation as *Peromyscus* mice are reported to be effective at removing and damaging infesting larvae [43]. Larval loss could also be partially explained by the variable attachment ability of larvae related to variation in larval activity during the infestation period and in varying capacity of individual larvae to attach to and feed on mice. Nilsson and Lundqvist [46] reported that ticks that do not find suitable feeding sites can actively leave the host or passively fall off due to host movements and larval attachment rates of less than 50% on mice are not uncommon in the literature [51]. A low rate of larval attachment could be explained by the fact that no device or procedure was used to restrain mouse movements or grooming behavior, potentially decreasing the attachment success of larvae post-anesthesia [52, 53]. Visual inspection of mice could also have underestimated the number of attached larvae as ticks may have attached in locations where it was hard to see them (e.g. in the dense fur on their backs or between their toes). Nevertheless, the low rate of attachment does not affect the conclusion of this study, given that the application of the same infestation technique in each group, and of a standardized observation method, ensured that control and treatment groups remained comparable.

## Conclusions

This study showed that fluralaner is effective at killing *I. scapularis* ticks that infest *Peromyscus* mice, a natural reservoir host of LD. This is a first step towards potential use of fluralaner in baits to treat wild rodents as an intervention to reduce LD risk in North America. However, more research is needed to better understand duration of efficacy, pharmacokinetics and toxicology of fluralaner in wild rodents in order to evaluate treatment efficacy, safety and predictability. The efficacy of smaller and shorter treatments when determining a treatment dose and refilling frequency for baits targeting wild rodents like *Peromyscus* mice in an intervention setting should also be considered. Further pharmacological research on mice in the laboratory setting and field trials in wildlife could help address some of these questions.

## Data Availability

The datasets generated during and/or analysed during the current study are available from the corresponding author upon reasonable request.

## Abbreviations

AUC: area under the curve
C_p_: plasma concentration
GLM: generalized linear model
LD: Lyme disease
SD: standard deviation
SE: standard error
